# Supplementary material for: Surface Microbiome Profiling of Dental Elevators Using Third‐Generation Sequencing: Implications for Infection Control in Dental Practice
Source: Microbiologyopen. 2025 Dec 8;14(6):e70178. doi: 10.1002/mbo3.70178 (PMC12685768; doi:10.1002/mbo3.70178)
Supplement: Supplementary file 1 — customer_backup. [file MBO3-14-e70178-s001.zip › customer_backup/customer_backup/function_analysis/KEGG/allsample/K2species/allsample.reabundance_Class2Phylum.txt.pdf]

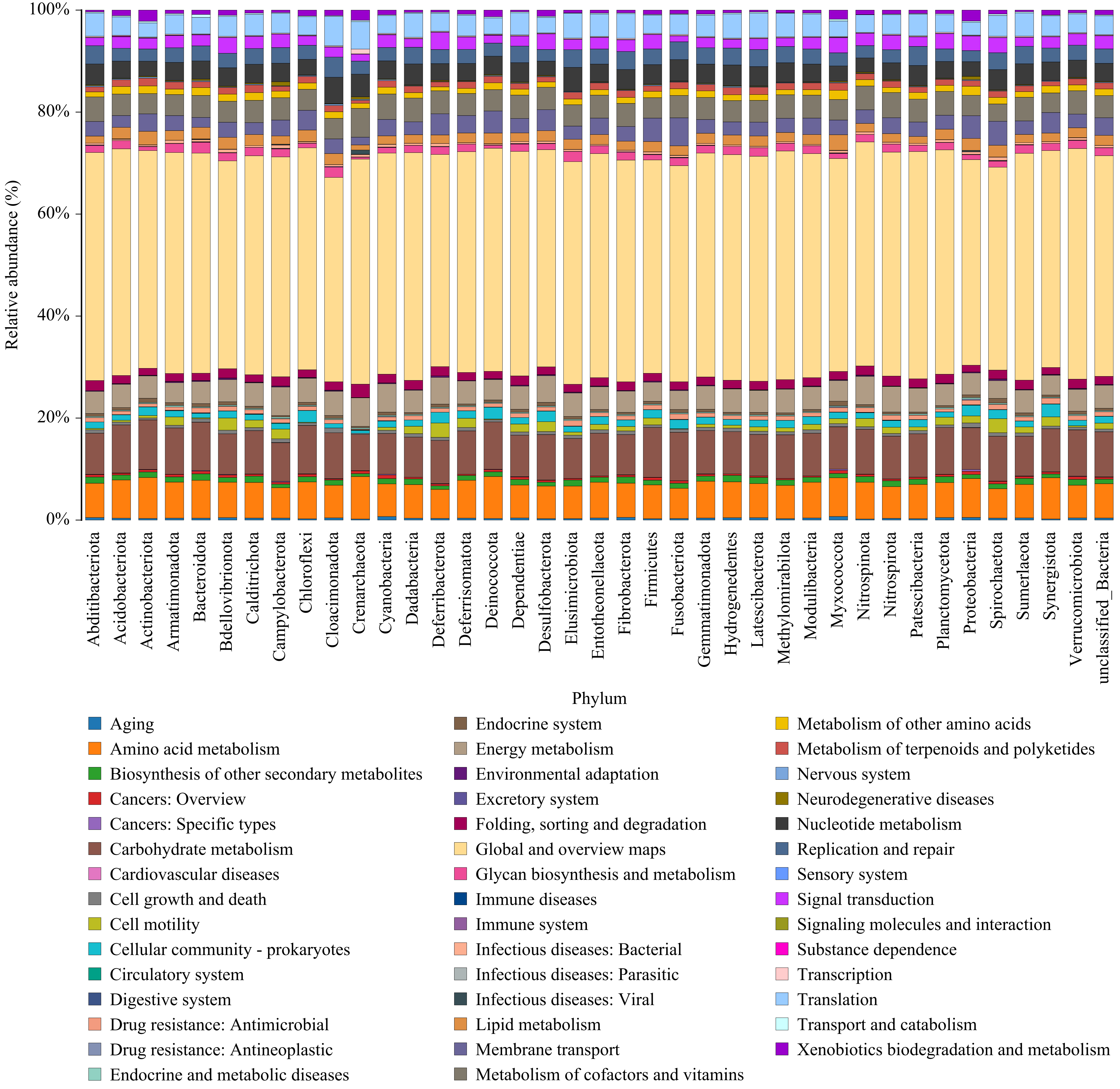

100%
80%
60%
40%
20%
0%
Relative abundance (%)
Abditibacteriota
Acidobacteriota
Actinobacteriota
Armatimonadota
Bacteroidota
Bdellovibrionota
Calditrichota
Campylobacterota
Chloroflexi
Cloacimonadota
Crenarchaeota
Cyanobacteria
Dadabacteria
Deferribacterota
Deferrisomatota
Deinococcota
Dependentiae
Desulfobacterota
Elusimicrobiota
Entotheonellaeota
Fibrobacterota
Firmicutes
Fusobacteriota
Gemmatimonadota
Hydrogenedentes
Latescibacterota
Methylomirabilota
Modulibacteria
Myxococcota
Nitrospinota
Nitrospirota
Patescibacteria
Planctomycetota
Proteobacteria
Spirochaetota
Sumerlaeota
Synergistota
Verrucomicrobiota
unclassified_Bacteria
Phylum
Aging
Amino acid metabolism
Biosynthesis of other secondary metabolites
Cancers: Overview
Cancers: Specific types
Carbohydrate metabolism
Cardiovascular diseases
Cell growth and death
Cell motility
Cellular community - prokaryotes
Circulatory system
Digestive system
Drug resistance: Antimicrobial
Drug resistance: Antineoplastic
Endocrine and metabolic diseases
Endocrine system
Energy metabolism
Environmental adaptation
Excretory system
Folding, sorting and degradation
Global and overview maps
Glycan biosynthesis and metabolism
Immune diseases
Immune system
Infectious diseases: Bacterial
Infectious diseases: Parasitic
Infectious diseases: Viral
Lipid metabolism
Membrane transport
Metabolism of cofactors and vitamins
Metabolism of other amino acids
Metabolism of terpenoids and polyketides
Nervous system
Neurodegenerative diseases
Nucleotide metabolism
Replication and repair
Sensory system
Signal transduction
Signaling molecules and interaction
Substance dependence
Transcription
Translation
Transport and catabolism
Xenobiotics biodegradation and metabolism
